# Pulmonary adenocarcinoma possibly developed from the cut-end of small-sized adenocarcinoma in the lung periphery as recurrence 13 years after its wedge resection

**DOI:** 10.1186/s40792-017-0413-0

**Published:** 2018-01-03

**Authors:** Takaomi Hanaoka, Makoto Kurai, Mitsuyo Okada, Satoshi Ishizone, Fumitoshi Karasawa, Akira Iizuka, Meguru Ikeyama, Jun Nakayama

**Affiliations:** 1 Department of Thoracic Surgery, JA Nagano North Alps Medical Center Azumi Hospital, 3207-1, Ikeda-machi, Kitaazumi-gun, Nagano, 399-8695 Japan; 2 Department of Respirology, JA Nagano North Alps Medical Center Azumi Hospital, Nagano, Japan; 3 Department of Surgery, JA Nagano North Alps Medical Center Azumi Hospital, Nagano, Japan; 4 Department of Molecular Pathology, Shinshu University Graduate School of Medicine, Nagano, Japan

**Keywords:** Lung cancer, Solid nodule, Limited resection

## Abstract

**Background:**

It is a big topic for general thoracic surgery whether still curability can be obtained by limited resection for peripheral small-sized nodules of non-small cell lung cancer (NSCLC) in the current era of frequent computed tomography (CT) use. Accumulation of information on problematic cases would be meaningful for surgeons to select better surgical procedures.

**Case presentation:**

A 69-year-old man was pointed out an enlarged 2.1-cm solid nodule on the edge of staple line of the residual right upper lobe by chest CT. He had past history of the lung cancer surgery, wedge resection of the same right upper lobe 13 years ago. The pathological findings were 1.1-cm, p-TlbN0M0, p-stage IA2-adenocarcinoma. Thereafter, he received no adjuvant therapy. This time, the trans-bronchial lung biopsy revealed adenocarcinoma. After the completion lobectomy of the residual right upper lobe, the tumor was diagnosed as adenocarcinoma consistent with recurrence of small-sized adenocarcinoma in the lung periphery developed from the cut-end because of similarities between present and previous tumors on histopathology and p53-positivity.

**Conclusions:**

When limited resection has been performed for small-sized NSCLC presenting solid nodule on thin-slice CT images, long-term postoperative follow-up time will be necessary for monitoring, considering the possibility of cut-end recurrence.

## Background

In the current era of frequent use of CT device, opportunities increase to excise small-sized pulmonary peripheral non-small cell lung cancer (NSCLC). It is a big topic whether still curability can be obtained by limited resection for it [1–4], because the evident curative surgical method of NSCLC has been historically lobectomy only [5]. It is also important for future contingency plan to accumulate information of problematic cases after limited resection [3, 4]. Here, we report such a case that possibly developed cut-end recurrence delayed during long-term follow-up time after wedge resection of peripheral small-sized adenocarcinoma.

## Case presentation

**Fig. 1:**
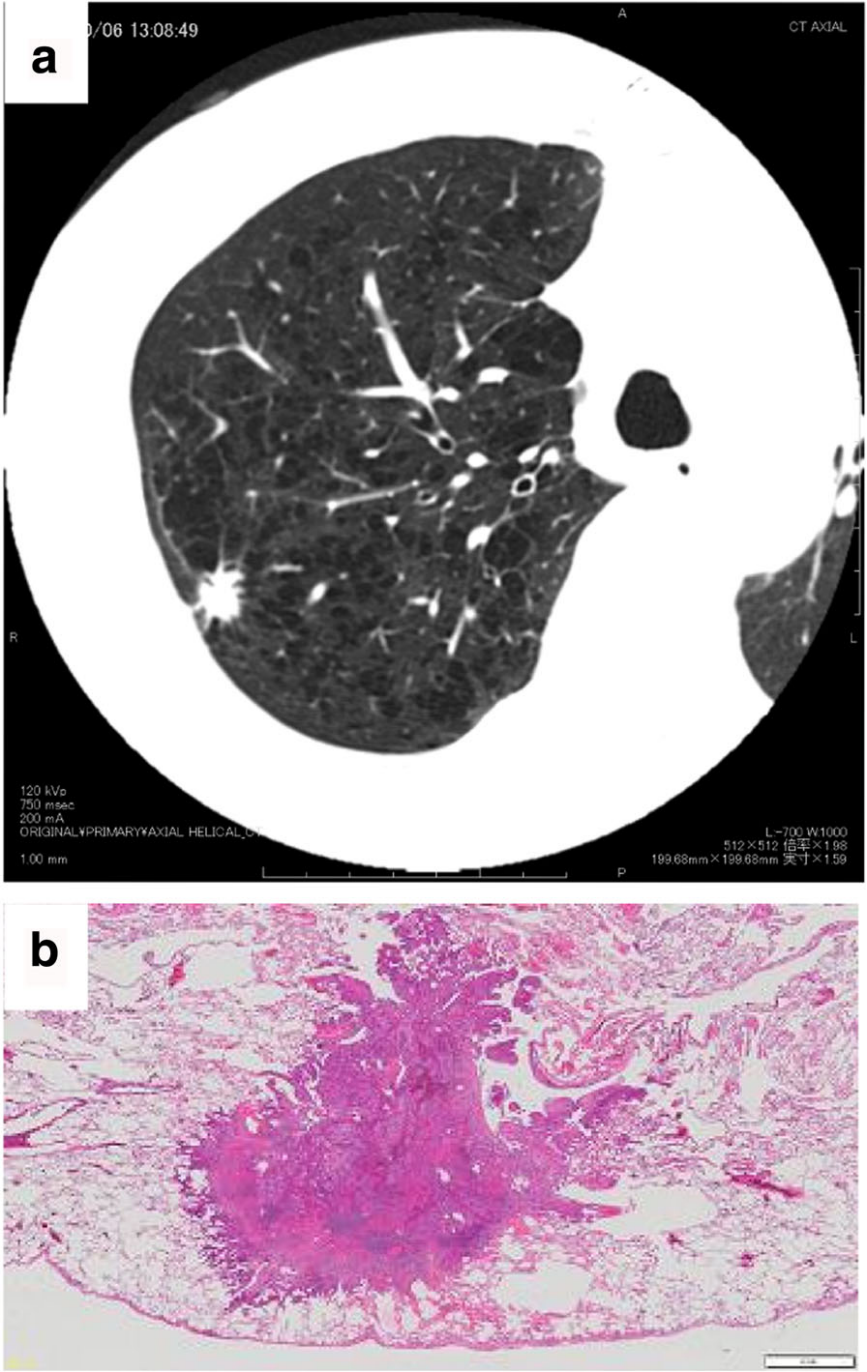
**a** Chest thin-slice computed tomography (TSCT) showing a 1.1-cm solid nodule in the right upper lobe. **b** Pathologic features of the nodule, showing adenocarcinoma (macroscopic image)

A 69-year-old male, ex-smoker, admitted to our hospital, presenting an enlarged solid nodule as cut-end recurrence in the residual right upper lobe without any signs and symptoms. On his past history, he underwent wedge resection of the right upper lobe by video-assisted thoracoscopic surgery (VATS) for a 1.1-cm solid nodule 13 years ago (Fig. 1). The pathological diagnosis was the second lung cancer, adenocarcinoma, mixed subtype, 1.1 cm, pl0, ly1, v0, p-T1bN0M0, p-stage IA2, complicated with emphysema. The intraoperative margin cytology both from the cut-end and from the cartridge of auto-suturing device showed negative findings. Epidermal growth factor receptor (EGFR) gene of the lesion was wild type. Further 4 months before that, he had undergone the contra-lateral left upper lobe lobectomy. The pathological diagnosis was the first lung cancer, adenocarcinoma, mixed subtype, 1.9 cm, pl1, ly1, v1, p-T2aN1M0, p-stage IIB. The EGFR gene was wild type. From these clinical courses, he had been diagnosed with bilateral synchronous double primary lung adenocarcinomas. After that, he could not receive any chemotherapy or any other medications for cancer, because of repeated acute pancreatitis.

**Fig. 2:**
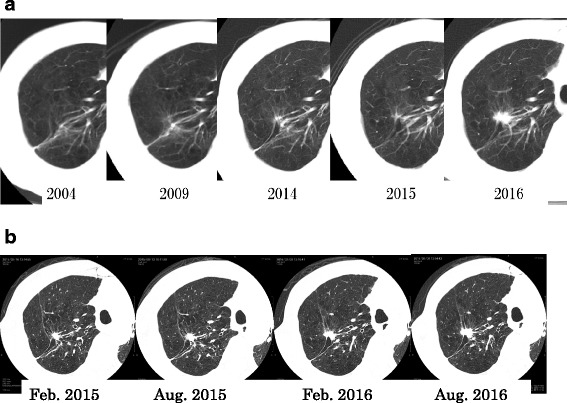
**a** Radiographic time course on conventional computed tomography (CT) images (0.8-cm slice) for 13 years. **b** Radiographic time course on thin-slice CT images (0.1-cm slice) for the last 2 years

**Fig. 3:**
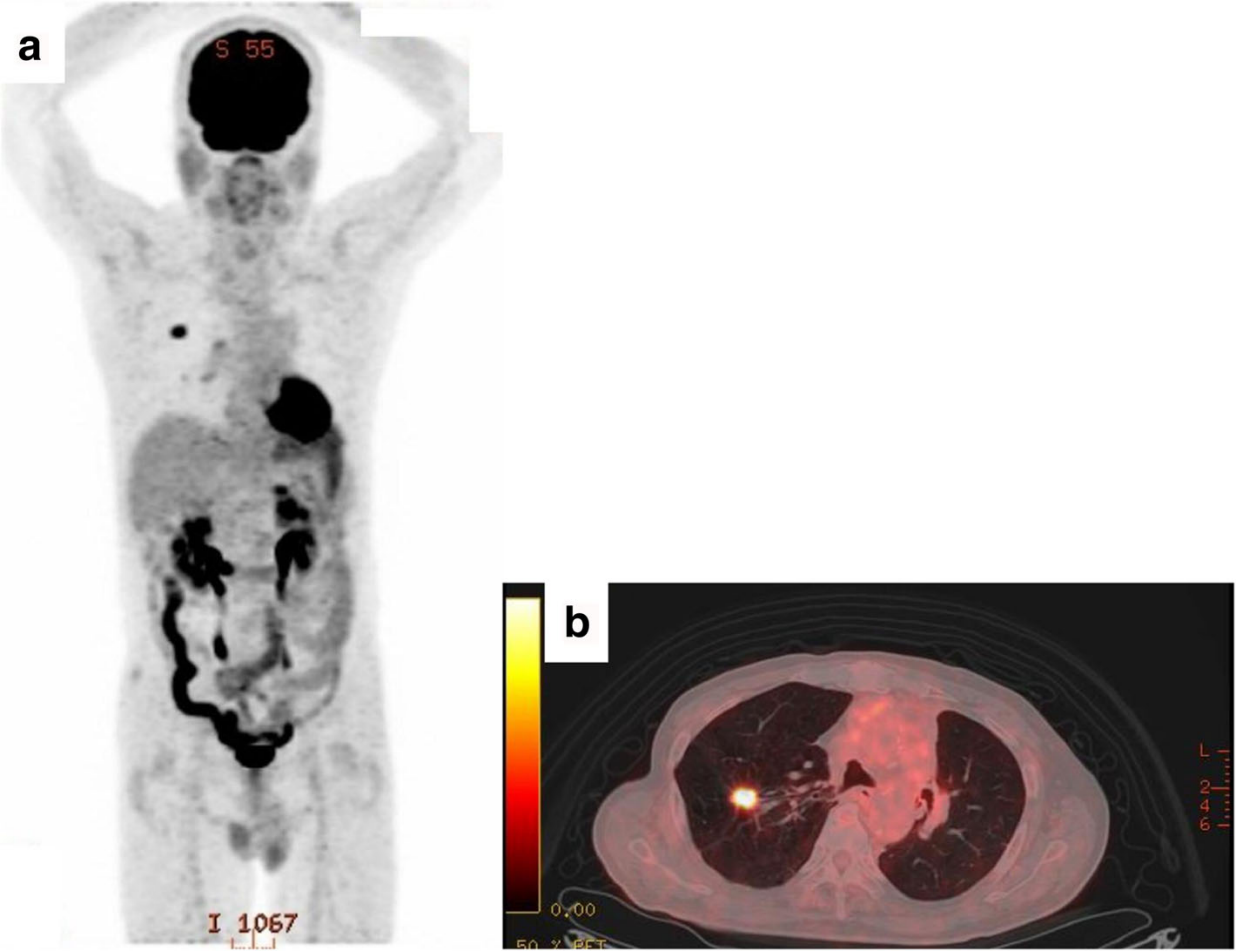
**a** Positron emission tomography (PET) showing accumulations of fluoro-deoxy-glucose (FDG) with the maximum standard uptake value of 7.2 in the residual right upper lobe and that of 2.6 in the right hilum. **b** PET/computed tomography (CT) showing accumulation of FDG in the solid nodule on this cut-end

**Fig. 4:**
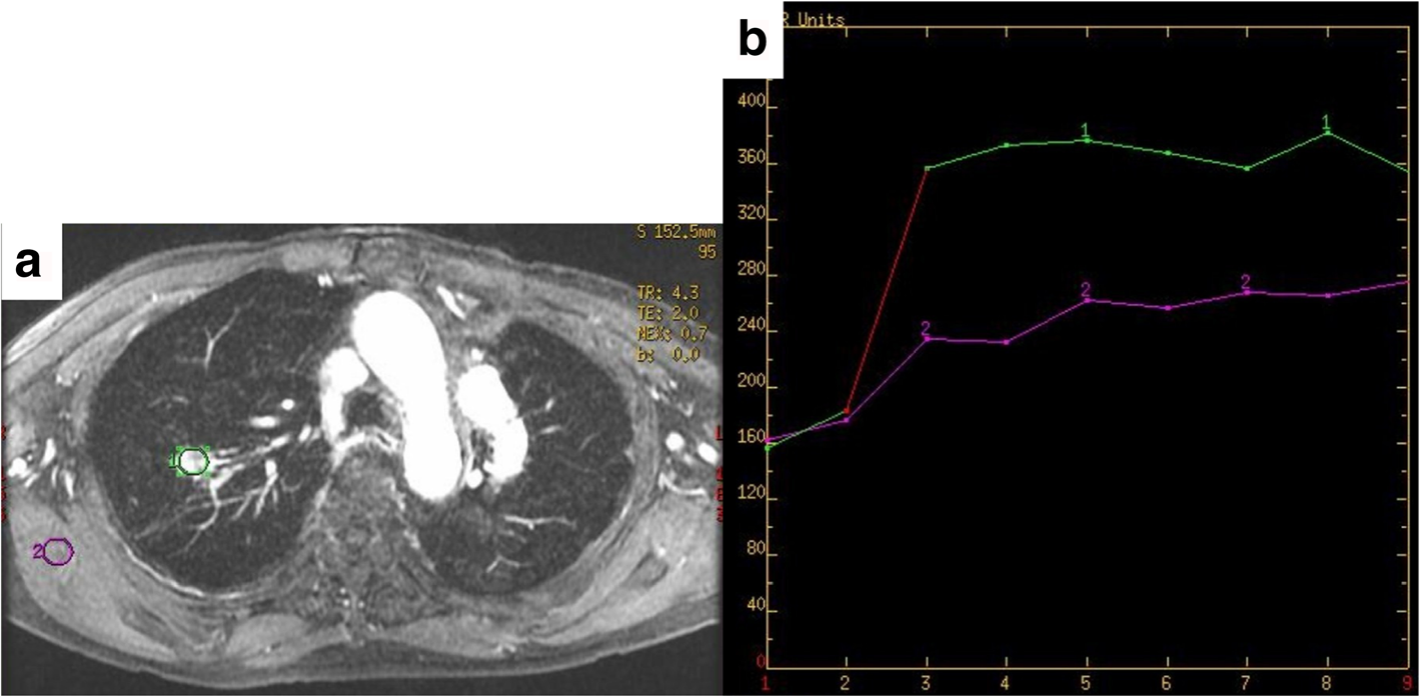
**a** Dynamic magnetic resonance imaging showing contrast enhancement effect in the solid nodule on the cut-end of the residual right upper lobe. **b** Graph showing time-intensity curve compared to normal muscular tissue of the chest wall

**Fig. 5:**
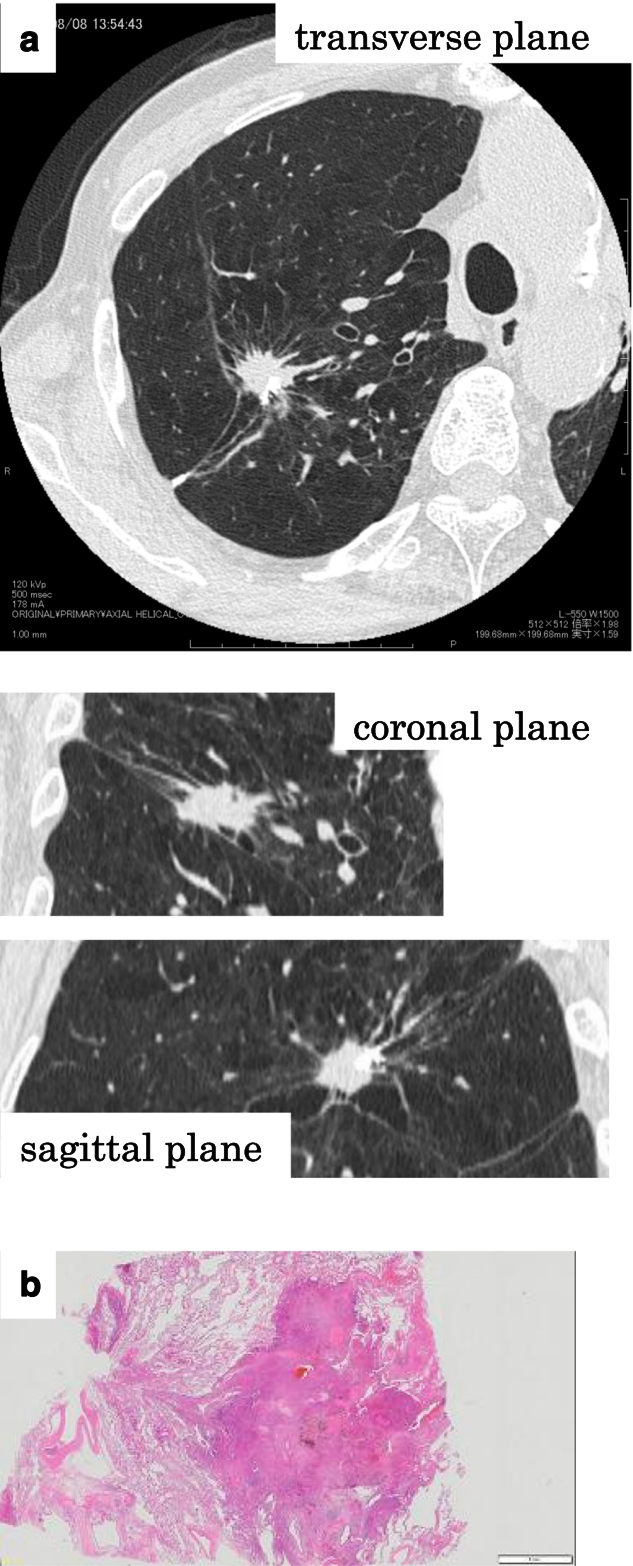
**a** Radiographic estimation by multi-planar reconstructions (transverse, coronal, and sagittal views) on thin-slice CT images (0.1-cm slice) before reoperation. **b** Pathologic features of the cut-end lesion of the residual right upper lobe, showing adenocarcinoma (macroscopic image)

**Fig. 6:**
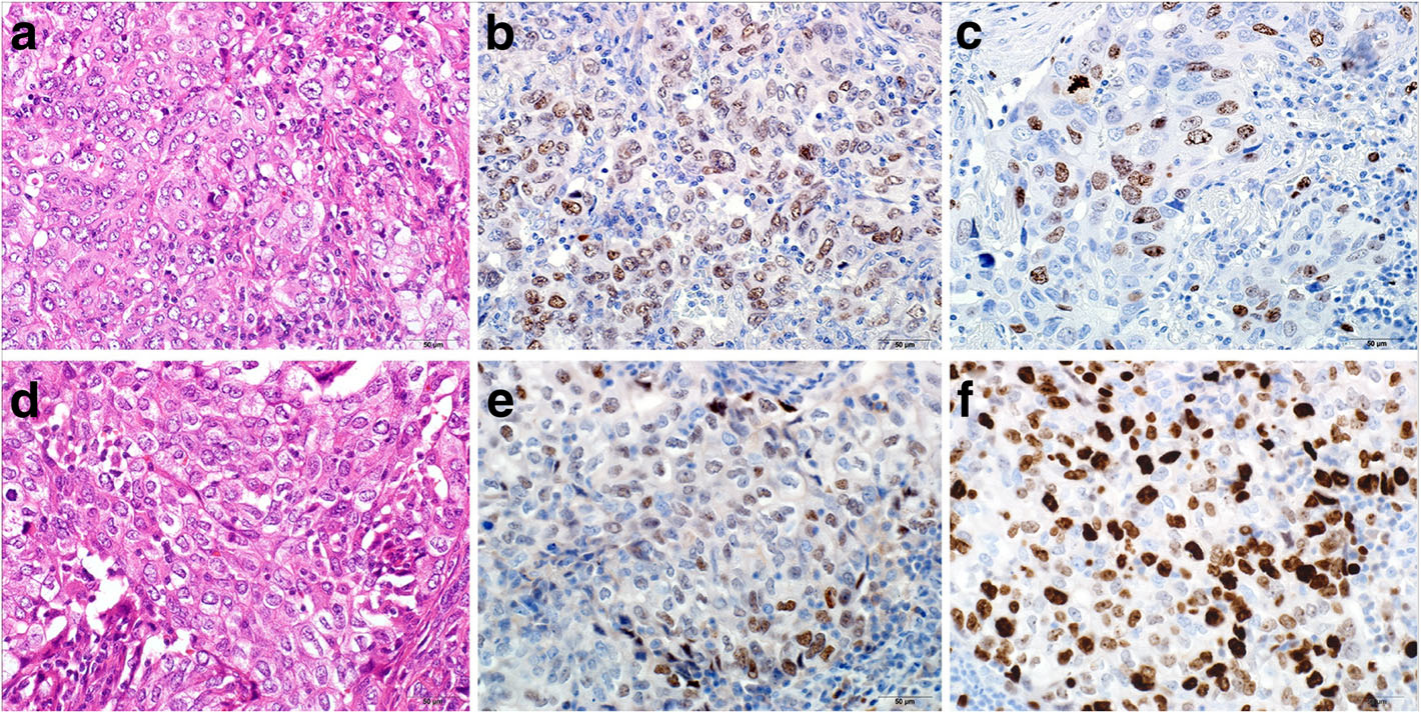
The upper images showing higher magnificent microscopic images of the primary tumor in the right upper lobe wedgely resected 13 years ago. The lower images showing those of the cut-end lesion in the residual right upper lobe. From left to right, hematoxylin and eosin staining (**a**, **d**) and immunostaining for p53 (**b**, **e**) and Ki-67 (**c**, **f**)

After 13 years from the last operation, chest thin-slice CT (TSCT) showed a non-calcified solid nodule with irregular margin and spicula formation, which enlarged to 2.1 cm on the end of staple lines of the residual right upper lobe (Figs. 2 and 5a). Other CT images concomitantly also showed another 2.1-cm pure ground-glass nodule (pGGN), in the neighboring interlober fissure of the right middle lobe, and some multiple subcentimeter-sized pGGNs in allover the lung field. Retrospectively viewing, the solid nodule only enlarged in the cut-end from the past 1 year ago, but it did not show any change before that (Fig. 2). Other pGGNs had no change of their size on TSCT images and were suspicious multiple atypical adenomatous hyperplasias. Positron emission tomography/CT findings showed abnormal accumulation only in the solid nodule on the cut-end (standardized uptake value [SUV] max 7.2), right hilar lymph node (SUV max 2.6), and no accumulation in any other pGGNs (Fig. 3). Dynamic magnetic resonance imaging of the solid nodule showed contrast enhancement effect (Fig. 4). The trans-bronchial biopsy for the solid nodule revealed adenocarcinoma, and it was clinically diagnosed as the cut-end recurrence. During these courses, the values of serum tumor markers (CEA and CYFRA) progressed almost within normal limits. By mini-thoracotomy, we performed completion lobectomy of the residual right upper lobe and wedge resection of the right middle lobe with ND1b. The final pathologic findings of the resected specimen diagnosed by two pathologists (MI and JN) were as follows: the solid nodule was adenocarcinoma, right upper lobe, mixed subtype (lepidic growth 10%), pT1c, 3.0 cm, pl0, ly1, v2, D0, E0, PLC-pre(-), R0, pm0. The histopathology of tumor was similar to that of the previously resected specimen 13 years ago (Figs. 5 and 6). The EGFR gene was also wild type. The pGGN of the right middle lobe was minimally invasive adenocarcinoma, pT1a, 2.0 cm (invasion 0.1 cm), pl0, ly0, v0. There was single positive loco-regional lymph node metastasis in the right hilum, #11s, pN1. Postoperative course was well, and he has no any recurrent lesions with good performance status after the last resection.

In order to examine the possibility of this tumor as recurrence of cut-end tumor, immunohistochemical comparisons between the present and primary tumors of the right upper lobe were carried out using p53 (clone PAb1801) and Ki-67 (clone MIB-1) antibodies [6, 7]. As results, p53 was positive for both tumors (Fig. 6b, e). For Ki-67, MIB-1 index of primary tumor was 30–50%, whereas that of the present tumor was less than 80% (Fig. 6c, f). Taken together, the similarities of histopathology and p53 expression patterns between both tumors suggested that the present adenocarcinoma could be developed from the cut-end of small-sized adenocarcinoma in the lung periphery as recurrence 13 years after wedge resection.

### Discussion

In the case of wedge resection, attention for complete resection was focused on the shortest distance from the cut-end to the tumor margin. The margin/tumor size ratio (M/T) and intraoperative margin cytology has been shown to relate with postoperative recurrence and survival rate [4]. This case showed negative margin cytology, but M/T ratio = 0.8 cm/1.1 cm < 1, and positive lymphatic invasion (ly1), which suggest an insufficient surgical margin. Therefore, cutters might dissect the margin of cancerous lesion, which was not detected by TSCT images, and then, minute amount of cancer cells had left behind, to regrow carcinoma tissue after a very long period of time. This main cause may be involvement of spread through air spaces (STAS) [8], which will be undetectable by any radiographic estimations. Other causes will be a possibility that one of multiple adenocarcinomas might be present originally in the vicinity of the cut-end and metachronously grow and be another one of single focal metastasis from the first adenocarcinoma of the precedent contra-lateral lung. However, these frequencies of the latter two causes are improbable. Regarding both the primary lesion of the right upper lobe and the cut-end recurrent lesion, these TSCT findings were common as solid nodule (Figs. 1 and 5), the histopathology of both tumors was similar (Fig. 6), and these EGFR gene mutations were both wild type. In addition, based on immunohistochemical analysis showing p53 nuclear mutant protein [6] and mitotic growth rate in cell cycle [7], we speculated that the tumor was possibly cut-end recurrence of residual tumor after resection 13 years ago rather than de novo occurrence associated with multicentric primary adenocarcinoma near the staple line. Considering why it took 13 years to recurrence, it is presumed generally that it will take several years to decades from the occurrence of cancer cells to the formation of tumor masses of a size detectable by diagnostic imaging. In our case, it is presumed that a very small number of cancer cells were left behind 13 years ago and then, that the tumor mass formation with a size which can be detected by diagnostic imaging was achieved by similar natural process of tumor formation.

Recently, since c-T category or malignant grade was sub-classified according to the size of solid component within small-sized nodule on TSCT images [9, 10], surgical procedures of NSCLC presenting solid nodule should be carefully selected by comprehensive estimation under the principle of standard lobectomy [5]. When firstly choosing limited resection for small-sized NSCLC cases with high-grade lesions, long-term follow-up is necessary in mind for postoperative observation by using TSCT, which can effectively capture delicate change of cut-end status in lung parenchyma.

## Conclusions

Small-sized pulmonary adenocarcinoma, presenting solid nodule on TSCT images, can recur on the cut-end after the wedge resection. In such cases, it is desirable to make a policy of completion lobectomy as a contingency plan within tolerable conditions of pulmonary function and medical comorbidities.

## Abbreviations

CT: Computed tomography
EGFR: Epidermal growth factor receptor
M/T: Margin/tumor size ratio
NSCLC: Non-small cell lung cancer
pGGN: Pure ground-glass nodule
SUV: Standardized uptake value
TSCT: Thin-slice computed tomography
